# Family planning in conflict: results of cross-sectional baseline surveys in three African countries

**DOI:** 10.1186/1752-1505-5-11

**Published:** 2011-07-13

**Authors:** Therese McGinn, Judy Austin, Katherine Anfinson, Ribka Amsalu, Sara E Casey, Shihab Ibrahim Fadulalmula, Anne Langston, Louise Lee-Jones, Janet Meyers, Frederick Kintu Mubiru, Jennifer Schlecht, Melissa Sharer, Mary Yetter

**Affiliations:** 1 RAISE Initiative, Heilbrunn Department of Population and Family Health, Mailman School of Public Health, Columbia University, New York, USA; 2 American Refugee Committee, Minnesota, USA; 3 Save the Children, Connecticut, USA; 4 International Rescue Committee, New York, USA; 5 RAISE Initiative/Marie Stopes International, London, UK; 6 CARE International, Georgia, USA; 7 Marie Stopes Uganda, Kampala, Uganda

## Abstract

**Background:**

Despite the serious consequences of conflict for reproductive health, populations affected by conflict and its aftermath face tremendous barriers to accessing reproductive health services, due to insecurity, inadequate numbers of trained personnel and lack of supplies. Family planning is often particularly neglected.

**Methods:**

In six conflict-affected areas in Sudan, northern Uganda and the Democratic Republic of Congo, household surveys of married or in-union women of reproductive age were conducted to determine baseline measures of family planning knowledge, attitudes and behaviors regarding contraception. Health facility assessments were carried out to assess baseline measures of family planning services availability. Data were double-entered into CSPro 3.2 and exported to SAS 9.2, which was used to calculate descriptive statistics. The studies' purposes were to guide program activities and to serve as a baseline against which program accomplishments could be measured.

**Results:**

Knowledge of modern contraceptive methods was low relative to other sub-Saharan African countries, and use of modern methods was under 4% in four sites; in two sites with prior family planning services it was 12% and 16.2%. From 30% to 40% of women reported they did not want a child within two years, however, and an additional 12% to 35% wanted no additional children, suggesting a clear need for family planning services. The health facilities assessment showed that at most only one-third of the facilities mandated to provide family planning had the necessary staff, equipment and supplies to do so adequately; in some areas, none of the facilities were prepared to offer such services.

**Conclusions:**

Family planning services are desired by women living in crisis situations when offered in a manner appropriate to their needs, yet services are rarely adequate to meet these needs. Refugee and internally displaced women must be included in national and donors' plans to improve family planning in Africa.

## Background

Conflicts and their aftermath can have dire consequences for reproductive health (RH). First, the preponderance of political emergencies occurs in the world's poorest nations, where the population's pre-conflict health is often already suboptimal [1]. Second, such crises bring sharply decreased access to services in a context of intensified threats. As health systems collapse and people flee in search of safety, access to health facilities that can offer safe delivery, provide emergency caesarean sections, or treat other complications of pregnancy and childbirth becomes limited or eliminated entirely. In many-even most-cases, women may be unable to obtain family planning methods during a time when few would choose to become pregnant if they had another option [2]. Safe abortion is often impossible to obtain even in peacetime, and during crises, women who have complications of unsafe abortions have no access to treatment [2]. Furthermore, women and girls may be raped or subject to other violence-as a strategy of war, due to the accompanying breakdown of order, or both-causing emotional and physical trauma and rendering them vulnerable to unwanted pregnancy and STIs, including HIV [2].

Despite these challenges, the provision of RH services to populations affected by armed conflict was long overlooked in traditional humanitarian response to complex emergencies [2]. Starting in the mid-1990s, however, the international community began to acknowledge the systematic absence of reproductive health services for these populations [3,4]. A 1994 report by the Women's Refugee Commission first documented this pervasive lack of attention to RH for refugees and internally displaced persons (IDPs), and its implications [4]. Although various human rights documents protect the right of all people to comprehensive reproductive health care [5], the 1994 International Conference on Population and Development (ICPD) and the Fourth World Conference on Women formally and specifically recognized this right of refugees and IDPs [6,7]. At ICPD, key agencies, including the United Nations Population Fund (UNFPA), the United Nations High Commissioner for Refugees (UNHCR), and the World Health Organization (WHO), committed to addressing this issue [7].

Subsequently, non-governmental organizations and national and local government authorities also began to acknowledge this need and examine ways to address it [1,8]. The Inter-agency Working Group on Reproductive Health in Crisis Situations (IAWG), comprised of UN agencies, humanitarian organizations, academic institutions and donors, was formed in 1995 [9]. In 1999, IAWG released the widely-used *Reproductive Health in Refugee Situations: An Inter-agency Field Manual *[7], a practical, concise guide to reproductive health programming for humanitarian workers, including health and other workers not expert in the topic.

After a decade and a half of sustained attention to the topic, much progress has been made in the field of RH in crisis settings in terms of policy, guidance and practice. For example, the Minimum Initial Services Package (MISP), a programmatic piece of the *Field Manual *that offers guidance on a suite of reproductive health services to be implemented during the earliest stages of an emergency, has been adopted into the *Sphere Humanitarian Charter and Minimum Standards in Disaster Response*. IAWG recently released a 2010 update to its 1999 field manual, now titled *Inter-agency Field Manual on Reproductive Health in Humanitarian Settings *[9]. A number of other guides have been developed that directly relate to the various RH services for conflict-affected populations, or that can be adapted for use in such a context: *Field-Friendly Guide to Integrate Emergency Obstetric Care in Humanitarian Programs; Family Planning: A Global Handbook for Providers; HIV Prevention and Control: A Short Course for Humanitarian Workers; *and *Clinical Care for Sexual Assault Survivors *[10-13]. In addition, stakeholders at many levels have had the opportunity to share field results and experiences at three conferences specific to RH in conflict and at annual IAWG meetings [14-17].

Furthermore, inroads have been made toward integrating RH into humanitarian response: for example, funds for reproductive health were included during the initial humanitarian response to the earthquake in Haiti in January 2010 [18].

Yet despite these important successes most conflict-affected women still do not have adequate access to RH services, and family planning services are often particularly neglected. A 2004 global evaluation of 10 years of attention to the reproductive health of populations affected by crisis concluded that most people affected by conflict still lack adequate RH care; refugees, camp populations and those living in stable settings had better access to care than did internally displaced people, non-camp populations and those in insecure areas, however. Regarding family planning, the evaluation found that where available at all, methods offered were frequently limited to oral contraceptives and condoms. Long-term and permanent methods were rarely offered, and for all methods, supplies were not reliable [19].

Program reports at conferences and meetings illustrate that gaps in services persist even now [14-16], but filling these service gaps presents a range of challenges. First, improving reproductive health services in conflict-affected countries presents all of the same difficulties faced by peaceful developing nations, plus a host of additional issues specific to conflict and post-conflict settings [20]. In general in conflict-affected areas, health systems have collapsed; communities are compromised; human resources are scarce and little attention is given to training for health workers who remain; health policy and management structures are in disarray; and logistical problems such as insecurity and damaged or nonexistent infrastructure circumscribe the movement of supplies, staff, and people in need of care [21,22].

Second, the long-term nature of conflict and its aftermath fits neatly into neither the mission of humanitarian groups nor development groups, meaning that neither sector is fully prepared to meet the reproductive health needs of refugees and internally displaced people. Humanitarian agencies are often structured to address the immediate needs of populations undergoing acute, short-term crises. Yet, emergencies-particularly political conflicts-are often far longer and more complex than this structure recognizes, and people may remain displaced for years, even decades. Even under UNHCR's relatively narrow definition of 'long-term displacement,' (25,000 people in exile for 5 years or more), 5.5 million people were in protracted refugee situations in 2009 [23]. Furthermore, protracted displacement situations are increasing in duration: in 1993, the average length of time a refugee lived in exile was 9 years; in 2003 it was 17 years [23]. Those affected by conflict may therefore live out a large portion of their lives in a context of displacement. This has significant implications for the needs of these populations and the nature of programs intended to meet these needs. Development agencies, on the other hand, work from a durable, systems-oriented perspective suited to the delivery of routine services over time to populations that include those experiencing protracted displacement, but such agencies may be ill-prepared to manage the challenges specific to working in insecure regions.

The RAISE Initiative and its partners work with populations and in regions identified by the 2004 global evaluation as especially under-served. In Africa, RAISE and its partners work in South Darfur (ARC), North Darfur (IRC), West Darfur (Save the Children-US), Southern Sudan (ARC), northern Uganda (MSU) and the Democratic Republic of Congo (CARE). The majority of residents served by these programs do not live in camps; at virtually all sites, insecurity has periodically affected delivery of services. This article describes the results of baseline studies of and the services at RAISE program sites in three countries. The studies' purposes were to guide program activities and to serve as a baseline against which program accomplishments could be measured. A further purpose of the studies, and a purpose of this article, is to document and disseminate data on family planning knowledge, attitudes and practices among population groups-conflict-affected, displaced women in this case-for whom such information is rarely available.

## Methods

The baseline measurement for each program is comprised of two components, a survey of women in the program's catchment area and an assessment of the health facilities serving them. Population-based household surveys of women of reproductive age and health facility assessments were conducted in six reproductive health program locations in three conflict-affected countries, Sudan, Uganda and the Democratic Republic of Congo (Table 1).

**Table 1:** Summary of survey methodology

	North Darfur	West Darfur	South Darfur	Southern Sudan	Northern Uganda	Eastern Congo
**Number of survey sites**	2	1	2	1	4	1

**Sample size**						
**Women of reproductive age**	929	816	922	626	1565	610
**Married and in union**	738	559	690	420	1238	558

**Data collection period**	Jun/Jul 2007	Apr/Jun 2008	Jul/Aug 2007	Nov/Dec 2007	Jul/Aug 2007	Nov 2007

The survey questionnaire was adapted from the *Reproductive Health Assessment Toolkit for Conflict-Affected Women *[24], developed by the US Centers for Disease Control and Prevention, which includes sections on demographics, safe motherhood, family planning, marriage and live-in relationships, sexual history, sexually transmitted infections, HIV/AIDS, gender-based violence, female genital cutting and emotional health. Program staff selected sections most appropriate to their context, and the survey instrument was translated into a total of five languages: in Uganda, Acholi and Lango; in DRC, French and Kiswahili; and in Darfur and in Southern Sudan, Arabic. The catchment areas for the six RH programs covered 11 geographic zones; stratified sampling ensured representativeness of each zone. Multi-stage sampling was employed within designated strata in which 25 to 30 villages were sampled with probability proportional to size. In each village, 25 to 30 households were selected. One woman of reproductive age was randomly selected from all eligible women within each selected household. Women aged 15-49 were eligible for inclusion in the sample in all programs except West Darfur; there, ever-married women aged 15-49 comprised the total survey sample. This analysis is restricted to women currently married and in union.

Women who gave oral informed consent completed face-to-face interviews, conducted in private by trained female interviewers. The earliest survey took place in June-July 2007 and the latest in June 2008. The surveys were approved by the Institutional Review Boards of Columbia University and institutional partners. Data were double-entered into CSPro 3.2 and exported to SAS 9.2, which was used to calculate descriptive statistics.

Facility assessments were conducted in all hospitals and health centers with which the programs would work, the quantity of which ranges from 2 to 21 facilities per program (Table 2). A team of MOH and NGO clinicians and managers trained in the assessment protocol comprised the assessment teams. Using a standardized instrument and methods of observation, interviews and record review, they evaluated physical infrastructure, human resources (number and type of posted and actual staff), infection prevention procedures, RH service readiness (availability and functionality of equipment and supplies) and RH services delivered in the prior 12 months. Assessments typically took one day in health centers and one to two days in hospitals. Assessments were carried out one to eight months prior to the surveys in the same areas.

**Table 2:** Facility assessments

	North Darfur	West Darfur	South Darfur	Southern Sudan	Northern Uganda	Eastern Congo
**Number of facilities assessed, by type**						
**Regional Hospital**	1	1	1	1	-	4
**Health Center**	2	7	1	1	8	17

**Data collection period**	Apr 2007	Oct/Nov 2007	Mar 2007	Mar 2007	Apr 2007	Nov/Dec 2007

## Results

### Population-based survey

The surveys' respondents were young, with mean ages between 27.3 and 28.9 years; poorly educated, with 29.2% to 58.1% reporting no schooling; and with a mean of 3.2 to 4.2 children (Table 3). Relative to sub-Saharan Africa, fewer women in the program areas were schooled and women at most sites had more children [25].

**Table 3:** Demographic profile of women married and in union

	North Darfur	West Darfur	South Darfur	Southern Sudan	Northern Uganda	Eastern Congo	Sub Saharan **Africa **[25]
**Mean age in years (SD)**	28.9 (7.6)	28.6 (6.93)	27.4 (7.1)	27.3 (6.8)	28.2 (7.8)	28.6 (8.5)	-

**Proportion with no education**	58.1	-	55.7	40.9	29.2	35.3	29.2

**Mean number of living children (SD)**	4.2 (2.7)	3.7 (2.8)	3.9 (2.7)	3.5 (2.3)	3.3 (2.3)	3.2 (2.3)	3.2

Current use of modern contraceptive methods among women in union was under 4% in four program areas, West and South Darfur, Southern Sudan and Eastern Congo (Table 4). The rate found in Eastern Congo, 3.2%, was lower than the 5.8% national level found in the 2007 Demographic and Health Survey. In West Darfur, CPR was 12%, slightly below the median CPR for 25 sub-Saharan African countries, 13.9% [25]. In northern Uganda, CPR was 16.2%, just under Uganda's national rate of 17.9% [26]. Oral pills were the most widely used method in all sites except Eastern Congo, where condoms were most popular, and northern Uganda, where injectables accounted for over half of all use.

**Table 4:** Modern contraceptive use among women of reproductive age married and in union

	North Darfur	West Darfur	South Darfur	Southern Sudan	Northern Uganda	Eastern Congo	Sub Saharan **Africa **[25]
**CPR (modern methods)**	2.3%	12%	1.7%	1.9%	16.2%	3.2%	13.9%

**Most common methods**							
**OCP**	1.9%	10.0%	1.6%	1.9%	1.9%	0.9%	
**IUD**	-	-	-	-	0.1%	-	
**Injection**	0.3%	1.0%	0.1%	-	9.0%	0.2%	
**Implant**	-	-	-	-	1.4%	-	
**Female sterilisation**	0.1%	-	-	-	1.4%	0.3%	
**Male condoms**	-	1.0%	-	-	2.4%	1.8%	

**Figure 1:**
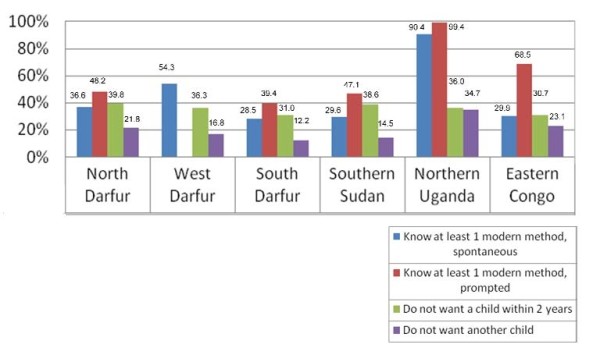
**Knowledge of and demand for family planning among women of reproductive age married and in union**.

Low use of modern contraception was matched by low levels of knowledge of contraceptive methods in most sites (Figure 1). Only in northern Uganda, where virtually all women were able to identify at least one modern family planning method when prompted, were levels of knowledge comparable to Africa overall, where the median level of prompted knowledge of at least one modern method was 92.6%. In the other program locations, prompted knowledge was far lower, ranging from 39.4% to 68.5% of respondents. Spontaneous knowledge of at least one modern method, a more rigorous test of knowledge, was very low in all sites except northern Uganda.

From 30.7% to 39.8% of women stated that they did not want a child in the next two years (spacers) and an additional 12.2% to 34.7% stated that they did not want any additional children (limiters). Relative to sub-Saharan Africa where the median proportion of limiters was 40.9%, fewer women in these conflict zones expressed a desire for limiting [25].

### Facility assessments

Of the 44 health facilities assessed, 38 were mandated to offer family planning services, with method choice dependent on facility type (Table 5). All but one of the mandated facilities reported having provided some form of family planning in the three months preceding the facility assessments. However, from zero to just over one-third of facilities were equipped with the necessary trained staff, equipment and supplies, including contraceptive commodities, to provide all mandated methods at the time of the assessment.

**Table 5:** Family planning supply

	North Darfur	West Darfur	South Darfur	Southern Sudan	Northern Uganda	Eastern Congo
**Number of facilities assessed**	3	8	2	2	8	21

**Number of facilities mandated to provide FP**	3	8	1	1	4	21

**Proportion of mandated facilities that provided any modern method in prior 3 months**	100%	100%	0	100%	100%	100%

**Proportion of mandated facilities with all required staff, equipment and supplies available (at time of assessment)**	33%	37.5%	0	0	25%	9.5%

## Discussion

The RAISE Initiative and its partner agencies in Africa, ARC, CARE, IRC, MSU and Save the Children-US, focus specifically on conflict-affected countries. Violent conflict disrupts health and education systems and distracts government attention from development efforts. Moreover, it undermines social cohesion, further hampering social and economic development. Of 182 countries listed in the United Nations Development Programme's 2009 Human Development Index, 24 are categorized as "Low Human Development." Of these 24, all but two (Timor-Leste and Afghanistan) are in sub-Saharan Africa and 14 have experienced conflict in the past 20 years [27]. Sudan, Uganda and DRC, the countries in which the programs discussed here are located, are ranked 150, 157 and 176, respectively, of the 182 countries in the Human Development Index [27].

The limitations of the studies must be recognized to fully understand and apply the results. The surveys and facility assessments were carried out over a one-year period, from June-July 2007 to June 2008, so while they are cross-sectional for each site, they reflect different time periods across sites. Surveys were administered in five languages; while translation and interviewer training were carefully conducted, such complexity may have affected the interviewers' or respondents' understanding of specific questions.

Given the level of poverty and conflict context in these program sites, in which respondents also reported low rates of school attendance, it is not surprising that modern method contraceptive prevalence among married women of reproductive age was found to be very low, under 4%, in four of the six program sites surveyed. In two sites, West Darfur and northern Uganda, prevalence rates were higher at 12% and 16.2%, respectively. Though high relative to other conflict sites and, arguably, relative even to sub-Saharan Africa in general, these rates are still low from a global perspective. Such low rates across all program sites are consistent with the known positive association between socio-economic status and use of modern family planning [28]. Low use of family planning was matched by poor knowledge of contraceptive methods, a consistent finding across all sites, with higher knowledge levels in the two higher prevalence sites.

It would be a mistake, however, to conclude that low use of family planning was due to lack of demand among women. In fact, demand for birth spacing was substantial, with 30% to 40% of women expressing their wish to not have a child in the next two years. Demand for limiting was expressed by an additional 12% to 35% of women across the sites. Instead, low prevalence can be attributed in large part to the poor supply of family planning services, with few facilities having all the required trained staff, equipment and supplies to provide good care, as the baseline health facility data make clear.

The results in northern Uganda, West Darfur, and Eastern Congo are instructive in examining the importance of consistent, trusted and comprehensive family planning service provision. In northern Uganda, Marie Stopes Uganda is the RAISE partner providing reproductive health services. Marie Stopes, a well-known and respected global reproductive health organization, has been operational in the north since 2002, providing the full range of family planning methods through clinics and outreach programs and offering extensive community education. Though northern Uganda is indeed a conflict zone, it is also part of a nation with a stable government and positive economic growth [29]. The survey results showing a contraceptive prevalence rate of 16.2%, the highest of all these conflict zones, and almost universal spontaneous knowledge of at least one family planning method highlight the importance of organizational commitment and consistent services.

In West Darfur, RAISE partners with Save the Children-US, which began programming, including family planning, in 2004. Thus, while the survey served as a baseline measure for the newly initiated program, its finding of 12% prevalence of modern methods in fact reflected some four years of community mobilization, community education and family planning service provision. Save the Children-US places high priority on working closely with communities to ensure that its programs meet their needs and to engender and earn the communities' trust. Through this approach, it was able to introduce family planning appropriately. Its programs serve both camp and non-camp populations and the survey showed higher family planning knowledge and use in camps, consistent with trends noted in the global evaluation.

In Maniema Province of Eastern Congo, CARE began a family planning project in partnership with the Ministry of Health in the province in 2004, and in August 2006, its mid-term evaluation found a modern contraceptive prevalence rate of 9% [30]. The project ended in March 2007, after which supplies and staff time available to support, manage and supervise education and service activities were limited. The baseline survey for the RAISE program was carried out in November 2007, and modern method prevalence was found to be 3.2%. Knowledge of family planning methods, desire to space children and desire to limit childbearing were, however, relatively high compared to other sites.

The low contraceptive prevalence rates found at baseline in most program sites were not surprising; on the contrary, the identified need for reproductive health services drove the selection of sites at the start of the Initiative. Prior program experiences among the partners and in the sites suggest that family planning education and a broad range of methods, provided in a manner appropriate to the local context and grounded in the community, will influence knowledge of family planning, desired timing of children, desire for additional children and ultimately, voluntary use of effective contraceptive methods.

Despite the pronounced need for these services, the data from the baseline health facility assessments indicate that they were largely unavailable to the women in these areas. Inadequate funding is one reason for limited family planning programming: between 2003 and 2006, less than US$1.30 was disbursed per capita per year for reproductive health in 18 conflict-affected countries, of which less than 2% was for family planning [31].

However, conflict-affected countries are not alone in their inability to provide the family planning services their people need and want, particularly in Africa. A review of family planning in sub-Saharan Africa demonstrated little or no reduction in unmet need for family planning in the last decade and found that unmet need is actually higher than contraceptive prevalence in many countries [32]. It further found that even those countries with relatively high contraceptive prevalence-accomplished after slow growth over many years in many cases-experienced a notable reduction in their rate of progress in the 2000s.

## Conclusions

Family planning services are a critical means of meeting women's and men's health needs and human rights in all countries of the world, including those affected by conflict. Data show a demand for spacing and limiting births among women in these sites, just as elsewhere in Africa; however, in these sites, the demand has far outstripped the available services. To fill this gap, family planning programs must be strengthened in sub-Saharan Africa, and refugees and displaced people must be included in national and donors' health and development plans. Moreover, all parties must maintain a long-term perspective, particularly in conflict-affected states, since history shows that progress in meeting communities' reproductive health needs has been slow even in countries at peace.

## Competing interests

The authors declare that they have no competing interests.

## Authors' contributions

TM, JA, SEC, LLJ, FKM and JS conceptualized and designed the study. RA, JA, SEC, SIF, FKM and JS were involved in data collection and field supervision. TM, JA, KA, RA, SEC, SIF, AL, FKM and MS analyzed and interpreted the data. TM was the principal writer of the manuscript, and JA, RA, SEC, SIF, AL, LLJ, JM, MS and MY each offered substantial revisions. All authors read and approved the final manuscript.
